# Supplementary material for: Learning curve and surgical outcome of robotic assisted colorectal surgery with ERAS program
Source: Sci Rep. 2022 Nov 29;12:20566. doi: 10.1038/s41598-022-24665-w (PMC9709162; doi:10.1038/s41598-022-24665-w)
Supplement: Supplementary file 1 — Supplementary Information 1. [file 41598_2022_24665_MOESM1_ESM.zip › LSC Raw data-Ñ[▒K/133/N_ñw╝╨░O▒Kñσ.pdf]

W83 / W83

臺中榮民總醫院
手術室護理記錄

[redacted]

基本資料

- 室別： 開刀房-06室，手術科別： CRS，手術類別： 常規，日期： 2021/08/19
- 進入時間： 1405 ； 麻醉開始時間： 1415 ； Time Out時間： 1500 ； 劃刀時間： 1501 ； 手術結束時間： 1800 送出時間： 1820 ；
- 麻醉方式： GE
  診斷：A COLON CA cT3N2M0
  手術名稱：Laparoscopic right colectomy and anastomosis
  手術局部用藥：NIL
  術前預防性抗生素：
  - 抗生素名稱一： Veterin 2GM IVD由麻醉護理人員協助給予
  - 時間： 1450
  - 上次簽章: 林湘芸

  ，追加預防性抗生素：
- 手術醫師： 蔣鋒帆，黃偑霖，洪浚嚴，郭連雄
- 麻醉醫師： VS 李淑娟
-
  - 刷手護士A ： 童歐文，時間： 1405，接替刷手一： 劉懿潔，時間： 1500
  - 流動護士A ： 林湘芸，時間： 1405，流動護士B ： 何佳青，時間： 1405，接替流動一： 洪豐麟，時間： 1730

皮膚狀況

A-V Shunt： 無
術前： 完整
記錄者： 上次簽章: 林湘芸
術後： 完整
記錄者： 上次簽章: 洪豐麟

手術準備

手術臥式 單一手術臥式

-
  - 手術臥式： 仰臥
  - 使用附件： 溫毯 38 ℃，矽膠枕頭，其它： Silicon pad
  - 皮膚準備： 10%Aq Betadine，Hibiscrub，2% Chlorhexidine
  - 電燒： 使用電燒: 電極片位置： 其它： 迴路電極墊，記錄者： 上次簽章: 林湘芸

止血帶T： 無

植入物

- 植入物名稱/號碼/數量/部位：Covidne GIA/80-3.8x2/A-colon

，記錄者： 上次簽章: 洪豐麟

標本送檢

- 細菌培養 無
- 細胞學 無
- 冰凍切片 無
- 病理標本
  組織名稱一：A-colon
  ，
  組織名稱二：LN 213
- 截肢 無

標本交付 標本： 2 袋，標本： 0 盒，標本： 0 瓶，細胞學： 0 管，Culture Tube： 0 管，被交付醫師： R 郭連雄，

- 記錄者： 上次簽章: 洪豐麟

- 時間： 1750

傷口術後數料

傷口數料： 傷口數料： 無

- 請於圖上標示部位：

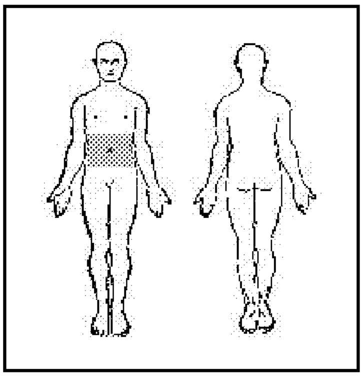

- 傷口數料： meport

導管

導管	天數	導管資訊	插入日期	插入時間	更換日期	拔除日期	拔除時間	拔除原因	預計拔除/更換	預計拔除/更換日期
Foley(1)	1	號碼:16 Fr.; 部位:膀胱;	2021/08/19	1435						
Epidural PCA(1)	1	部位:硬脊膜;	2021/08/19	1230						
A-line (1)	1	號碼:20 G; 部位:右手;	2021/08/19	1430						

用物計數

- 手術中出血量： minimal c.c.
- 手術中尿量： 400 c.c.
-
  - 其它： 無

用物計數： 多切口同時縫合

- 1.器械： 正確，2.縫針： 正確，3.刀片： 正確，4.紗布： 正確，5.腹部墊： 未使用，6.紗球： 未使用，7.PEANUTS ： 未使用，8.紗條： 未使用，9.絲帶： 未使用，10.針頭： 未使用，11.Vessel loop： 未使用，12.Boots： 未使用，13.Jelco： 未使用，14.棉條： 未使用，
  - 第一次：
    - 刷手護士 ： 劉懿潔
    - 流動護士： 上次簽章: 洪豐麟
    - 時間： 1735

  ,
  - 第二次：
    - 刷手護士 ： 劉懿潔
    - 流動護士： 上次簽章: 洪豐麟
    - 時間： 1745

  ,
  - 第三次：
    - 刷手護士 ： 劉懿潔
    - 流動護士： 上次簽章: 洪豐麟
    - 時間： 1755

備註

備註： R6 Olympus scope NO.6

- 記錄者： 上次簽章: 林湘芸
- 時間： 1430

填寫護理人員 洪豐麟

正式摘要

		W83 / W83				

doctorRank:B
